# Comparative effectiveness of group *v*. individual trauma-focused treatment for posttraumatic stress disorder in veterans

**DOI:** 10.1017/S0033291722001441

**Published:** 2023-07

**Authors:** Tobias R. Spiller, Or Duek, Eugenia Buta, Georgina Gross, Noelle B. Smith, Ilan Harpaz-Rotem

**Affiliations:** 1 Clinical Neurosciences Division, National Center for PTSD, VA Connecticut Healthcare System, 950 Campbell Avenue, West Haven, CT 06516, USA; 2 Department of Psychiatry, Yale University School of Medicine, 333 Cedar St, New Haven, CT 06510, USA; 3 Yale School of Public Health, Yale University, 60 College St, New Haven, CT 06510, USA; 4 Northeast Program Evaluation Center, VA Connecticut Healthcare System, 950 Campbell Avenue, West Haven, CT 06516, USA; 5 Department of Psychology, Yale University, New Haven, CT 06510, USA

**Keywords:** Posttraumatic stress disorder, CPT, PE, veterans, effectiveness

## Abstract

**Background:**

Cognitive processing therapy (CPT) and prolonged exposure (PE) delivered in an individual setting are efficacious and effective treatments for veterans with posttraumatic stress disorder (PTSD). Group CPT has been shown to be less efficacious than individual CPT, however, evidence regarding real-world effectiveness is limited.

**Methods:**

We conducted a retrospective, observational, comparative effectiveness study including veterans that received at least eight sessions of group CPT, individual CPT, or individual PE, and were discharged from PTSD residential treatment at the Department of Veterans Affairs between 1 October 2015, and 30 September 2020. PTSD symptom severity was assessed with the PTSD Checklist for DSM-5 (PCL-5) and treatments delivered in a group (CPT) or individual (CPT or PE) setting were compared at discharge and 4-month post-discharge follow-up.

**Results:**

Of 6735 veterans, 3888 [653 women (17%), median (IQR) age 45 (35–55) years] received individual and 2847 [206 women (7.2%), median (IQR) age 42 (34–54)] received group therapy. At discharge, improvement in PTSD severity was statistically greater among those treated individually (mean difference on the PCL-5, 2.55 (95% CI 1.61–3.49); *p* = <0.001]. However, the difference was smaller than the minimal clinically important difference of 7.9 points. The groups did not differ significantly at 4-month follow-up [mean difference on the PCL-5, 0.37 (95% CI −0.86 to 1.60); *p* = 0.551].

**Conclusion:**

Group CPT was associated with a slightly smaller reduction of PTSD symptom severity than individual CPT or PE in veterans at the end of residential treatment. There were no differences at 4-month follow-up.

Posttraumatic stress disorder (PTSD) is a chronic, debilitating psychiatric disorder affecting between 10 and 20% of U.S. veterans (Fulton et al., 2015; Gates et al., 2012; Marmar et al., 2015; Sundin, Fear, Iversen, Rona, & Wessely, 2010). Veterans with PTSD often suffer from additional mental and physical health conditions (Nichter, Norman, Haller, & Pietrzak, 2019; Seal et al., 2011), are more likely to have poor occupational functioning (Vogt et al., 2017), and to be without housing (Tsai & Rosenheck, 2015). Over the last two decades, efficacious and effective psychotherapeutic interventions for PTSD have been developed and tested in veteran populations (Kitchiner, Lewis, Roberts, & Bisson, 2019; Steenkamp, Litz, Hoge, & Marmar, 2015). The most robust evidence has emerged for individually delivered cognitive processing therapy (CPT) and prolonged exposure (PE), with both being superior to control treatments in randomized controlled trials (Steenkamp et al., 2015; Steenkamp, Litz, & Marmar, 2020), as well as effective in clinical practice (Maguen et al., 2021). In a direct comparison in non-veterans, both were found to be equally efficacious (Resick, Nishith, Weaver, Astin, & Feuer, 2002). Consequently, the Veterans Health Administration of the Department of Veteran Affairs (VA) currently recommends and provides individually-delivered CPT and PE as first-line treatments (Department of Veterans Affairs & Department of Defense, 2017).

Group psychotherapy proved to be only half as efficacious in reducing symptoms of PTSD than individual psychotherapy in a randomized clinical trial with active-duty military personnel (Resick et al., 2017). Nevertheless, robust evidence indicates that a variety of trauma-focused and non-trauma-focused group interventions efficaciously reduce PTSD severity in veterans (Resick et al., 2015; Sloan, Feinstein, Gallagher, Beck, & Keane, 2013, 2018; Steenkamp et al., 2015). This evidence is reflected in the Department of Veterans Affairs and Department of Defense, 2017 Clinical Practice Guideline for the Management of Posttraumatic Stress Disorder which recommends group psychotherapy for veterans with PTSD as a second-line treatment and only when individual treatments are not available or when a veteran has a strong preference for group psychotherapy (Department of Veterans Affairs and Department of Defense, 2017).

Despite large efforts to establish first-line evidence-based treatments for PTSD (i.e. individual psychotherapy) throughout the VA health care system (Karlin et al., 2010), thousands of veterans still participate in group therapy every year. Notably, evidence on the effectiveness of group therapy for veterans with PTSD in clinical practice is very limited. The only observational study reported a medium effect size (Cohen *d* = 0.38) for the reduction in PTSD symptom severity from pre to post treatment in veterans treated in a group setting (Castillo, Lacefield, Baca, Blankenship, & Qualls, 2014). This effect is smaller than the corresponding effect (Cohen *d* = 0.6) found in a randomized control study testing the efficacy of group CPT compared to individual CPT (Resick et al., 2015). However, the sample was limited to 291 women veterans, which is not representative of the veteran population treated for PTSD. Thus, it is unclear whether veterans receiving group CPT are in fact receiving a less effective treatment. The present study aimed to investigate the clinical-practice effectiveness of CPT group therapy compared to individual CPT or PE in reducing symptoms of PTSD in veterans treated in the VA health care system's residential PTSD treatment program.

## Method

### Participants and procedures

This comparative effectiveness study retrospectively analyzed the cohort of veterans who were discharged from VA PTSD residential treatment between 1 October 2015, and 30 September 2020. The VA PTSD residential treatment programs provide intensive treatment to address symptoms associated with PTSD in a residential setting with 24/7 supervision and support. Programs rely on a combination of various methods to assign a diagnosis, including structured and unstructured clinical interviews, chart reviews, historical diagnoses, and information from referring providers. Per VHA Directive 1162.02, admission criteria include: not meeting criteria for acute mental health or medical inpatient treatment, requiring a level of care higher than outpatient management (or no outpatient management is available), being capable of self-preservation and basic self-care, and not being an imminent risk to self or others. The latter is determined by the mental health clinician conducting the screening appointment prior to the admission. The VA PTSD residential treatment program is evaluated by the Northeast Program Evaluation Center, which collects information on demographics, PTSD symptom severity and treatment satisfaction for all veterans with a minimum admission length of three days. Self-report forms are completed by veterans in person at admission and discharge, and by mail four months following discharge. Upon discharge, clinicians submit a form indicating information about the residential treatment episode, such as type of treatment(s) and number of sessions received. Treatment was provided at 42 sites across the U.S. For this study the following inclusion criteria were set: (a) participants received at least 8 sessions of individual CPT or PE, or group CPT; (b) participants reported demographic data including data on race and ethnicity; (c) participants completed PTSD symptom assessment at least once at admission, discharge or follow-up. Participants with a PCL-5 score less than 30 at admission [indicative of no PTSD diagnosis(Blevins, Weathers, Davis, Witte, & Domino, 2015) were excluded from the analysis, based on the assumption of unreliable symptom reporting. The study protocol was approved by the VA Connecticut Healthcare System Institutional Review Board (IHR0014).

### Measures

Veterans self-reported demographic data on the admission form at admission to the residential PTSD program. Variable options for race included White, Black, American Indian/Alaskan, Asian, Pacific Islander, Other, and ethnicity included Hispanic, or Non-Hispanic. Trauma exposure was characterized by: (1) a binary item regarding combat trauma and (2) an item asking about additional traumatic life events (Military Sexual Trauma, Non-Military Sexual Trauma, Vehicle accident, Other accident, Victim of Violence (e.g. child abuse), Natural disaster, Other, and None). Symptoms of anxiety and depression at admission were quantified with the Generalized Anxiety Disorder-7 [GAD-7; (Spitzer, Kroenke, Williams, & Löwe, 2006) and the Patient Health Questionnaire-9 [PHQ-9; (Kroenke, Spitzer, & Williams, 2001), respectively. Both questionnaires use a 4-point Likert scale (ranging from 0 = ‘not at all’ to 3 = ‘nearly every day’), consist of seven (GAD-7) or nine (PHQ-9) items, and are self-report measures.

PTSD symptom severity was measured with the PTSD Checklist for DSM-5 [PCL-5; (Blevins et al., 2015) at admission, discharge, and follow-up. The PCL-5 is a self-report measure of the severity of 20 PTSD symptoms corresponding with the DSM-5 diagnostic criteria for PTSD. The severity of each symptom in the past month is rated on 4-point scale (ranging from 0 = ‘not at all’ to 3 = ‘extremely’) and summed to produce a total score with a possible range from 0 to 80, with higher scores indicating higher symptom burden.

Veteran satisfaction with progress towards personal recovery goals was quantified with a single question at admission, discharge, and follow-up. Possible response values ranged from 0 to 4, with higher scores indicating higher satisfaction. Satisfaction with provided care was assessed at follow-up with a single question (ranging from 0 = ‘Not at all satisfied’ to 4 ‘Completely satisfied’). Additional information about the treatment site, the modality of received treatment (CPT, PE, or group CPT), the length of stay in the treatment program, and the receipt of substance use disorder services while in residential PTSD treatment (yes/no) was obtained from forms completed by the therapists.

### Treatments

PE (Rothbaum, Foa, & Hembree, 2007) and individual CPT (Resick, Monson, & Chard, 2016) are manualized cognitive-behavioral interventions for PTSD that aim to address PTSD symptoms through alteration of cognitions and behaviors. Group CPT is a second-line treatment that includes many of the same elements of CPT but is delivered in a group format. All three treatment manuals were widely disseminated at the VA with a rigorous training and certification process (Chard, Resick, Monson, & Kattar, 2009; Resick, Monson, & Chard, 2010). Details about the training of the individual therapists and the VA-wide implementation program are outlined in detail elsewhere (Karlin et al., 2010). All 42 treatment sites included in this study provided evidence-based treatment for PTSD. However, the length of the programs, and the additional mental and physical health services offered varied across treatment sites.

### Statistical analysis

Differences between the baseline characteristics of the two treatment groups (group CPT and Individual CPT/PE) were assessed using Wilcoxon rank-sum test for continuous variables and χ^2^ test for discrete variables. We used a linear mixed-effects model to assess our primary outcomes, namely PTSD symptom severity differences between the two treatment groups at admission, discharge, and follow-up. Time (with three levels: admission, discharge, follow-up), treatment group (with two levels: individual and group) and their interaction were added as fixed effects. The model further included random intercepts for individuals and for treatment sites. To adjust for differences in demographic and socio-economic variables between the groups at baseline, the following variables were all included as categorical covariates: Woman (yes/no), Race [categorized as White, Black, and Other (American Indian/Alaskan, Asian, Pacific Islander, and Other)], Ethnicity (Hispanic/Non-Hispanic), combat-trauma (yes/no), military sexual trauma (yes/no), non-military sexual trauma (yes/no), vehicle accident (yes/no), other accident (yes/no), and substance use disorder treatment (yes/no). Age was entered as a continuous covariate. The covariance structure was specified as autoregressive with a lag of 1. The outcomes of interest were estimated marginal means with post-hoc contrast between treatment groups (Searle, Speed, & Milliken, 1980). Missing data were not imputed because mixed models can produce relatively robust estimates even when some individuals have missing observations if the data is missing at random (Detry & Ma, 2016).

Two sensitivity analyses were conducted to test the robustness of our results obtained for the primary outcomes. First, to test whether differences in PTSD symptom severity between the treatment groups at admission affected the results, we repeated the analysis including PCL-5 score at admission and its interaction with time as a covariate. Consequently, the dependent variable was PCL-5 score at discharge and follow-up. Second, to investigate how differences in treatment modalities influence the results, the analysis was repeated excluding individuals who received PE (see online Supplementary Materials).

The first secondary outcome, treatment satisfaction at admission, discharge, and follow-up, was tested with the same linear mixed model as the primary outcome. The other secondary outcome, satisfaction with provided care at follow-up, was also tested using a mixed linear model, with the same covariates except for time. Unadjusted *p* values are reported. All analyses were conducted using R statistical software version 4.0.3. The analyses were conducted between July and November 2021.

## Results

**Fig. 1.**
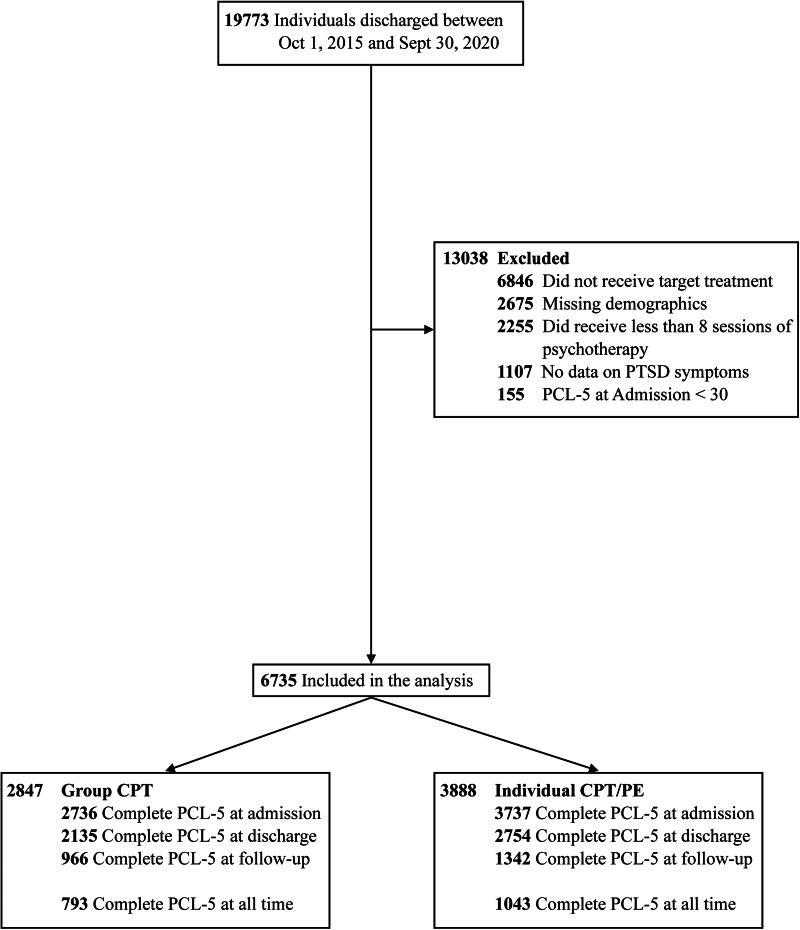
Flow of participants through the study.

Figure 1 depicts the flow of participants into and through the study. A total of 6735 veterans, 3888 in individual treatment and 2847 in group treatment, were included. Of these, 6473 (96%) reported complete data on PTSD severity at admission, 4889 (73%) at discharge and 2308 (34%) at 4-month follow-up. Participants in the individual treatment group more often identified as women, Black and Non-Hispanic, reported less combat, but more sexual trauma, stayed longer in treatment and were older than participants in group treatment (Table 1). The distribution of raw PCL-5 scores is shown in the supplement (online Supplementary Fig. S1).

**Fig. 2.**
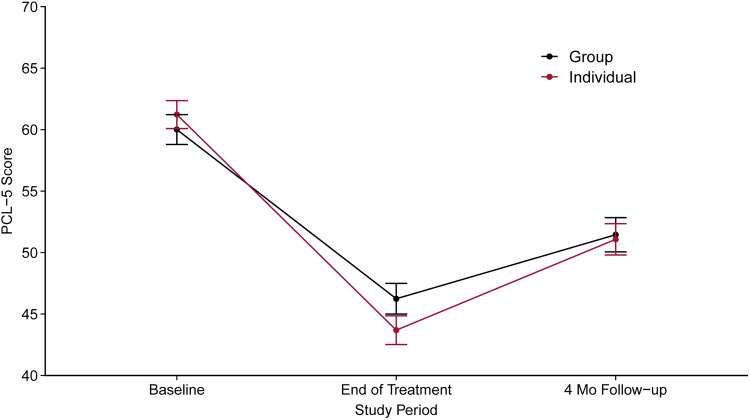
Estimated mean PCL-5 scores at all time points.

**Table 1.** Demographics and characteristics of participants by treatment condition

	Treatment condition, No (%) of patients^a^			
Variable	Overall (*N* = 6735)	Group CPT (*n* = 2847)	Individual CPT/PE (*n* = 3888)	*p* Value
Age, median (IQR), y	44 (35, 55)	42 (34, 54)	45 (35, 55)	<0.001
Female gender	859 (13)	206 (7.2)	653 (17)	<0.001
Race				<0.001
American Indian/Alaskan	303 (4.5)	134 (4.7)	169 (4.3)	
Asian	77 (1.1)	34 (1.2)	43 (1.1)	
Black	1730 (26)	689 (24)	1041 (27)	
Other	197 (2.9)	71 (2.5)	126 (3.2)	
Pacific Islander	82 (1.2)	60 (2.1)	22 (0.6)	
White	4346 (65)	1859 (65)	2487 (64)	
Ethnicity				<0.001
Hispanic	592 (8.8)	321 (11)	271 (6.9)	
Non-Hispanic	6134 (91)	2526 (89)	3617 (93)	
Education, median (IQR), y	13 (12, 14)	13 (12, 14)	13 (12, 15)	0.42
Baseline symptom severity, median (IQR)				
GAD-7^b^	16.0 (12.0, 19.0)	16.0 (12.0, 19.0)	16.0 (12.0, 19.0)	0.44
PHQ-9^c^	18.0 (14.0, 22.0)	18.0 (14.0, 22.0)	18.0 (14.0, 22.0)	0.72
Combat trauma	4892 (73)	2262 (79)	2630 (68)	<0.001
Additional trauma^d^				
Military sexual trauma	1680 (25)	467 (16)	1213 (31)	<0.001
Non-military sexual trauma	1286 (19)	427 (15)	859 (22)	<0.001
Vehicle accident	3320 (49)	1499 (53)	1821 (47)	<0.001
Other accident	1659 (25)	775 (27)	884 (23)	<0.001
Victim of violence	2319 (34)	962 (34)	1357 (35)	0.34
Natural disaster	1089 (16)	479 (17)	610 (16)	0.21
Other traumatic incident	3537 (53)	1533 (54)	2004 (52)	0.062
None	229 (3.4)	107 (3.8)	122 (3.1)	0.17
Therapy				
Prolonged exposure	1226 (18)	0 (0)	1226 (32)	
CPT individual	2662 (40)	0 (0)	2662 (68)	
CPT group	2847 (42)	2847 (100)	0 (0)	
Program completion	6420 (95)	2729 (96)	3691 (95)	0.077
Length of stay, median (IQR), d	51 (46, 60)	49 (46, 57)	52 (46, 62)	<0.001
SUD services	4554 (68)	1986 (70)	2568 (66)	0.001

Abbreviations: CPT, Cognitive processing therapy; GAD-7, Generalized Anxiety Disorder – 7 item; PCL-5, Posttraumatic Stress Disorder Checklist for DSM-5; PE, Prolonged exposure; PHQ-9, Patient Health Questionnaire – 9 item; SUD, Substance abuse disorder.

a Percentages have been rounded and may not total 100.

b Scores range from 0 to 21, with higher scores indicating worse symptoms. There were missing data resulting in the following sample sizes for this item: Overall, *n* = 4684; Group CPT, *n* = 1907; Individual CPT/PE *n* = 1736.

c Scores range from 0 to 27, with higher scores indicating worse symptoms. There were missing data resulting in the following sample sizes for this item: Overall, *n* = 4678; Group CPT, *n* = 1905; Individual CPT/PE *n* = 1732.

d Multiple answers could be given.

### Primary outcome

After adjustment for covariates, the mean PCL-5 score at admission for group treatment was 60.0 (95% CI 58.8–61.2) and 61.2 (95% CI 60.1–62.4) for individual treatment, equivalent to a small between-group difference of 1.22 (95% CI 0.36–2.08, *p* = 0.006) points in favor of individual treatment. At discharge, mean PCL-5 score for the individual treatment group was 43.7 (95% CI 42.5–44.9) and 46.2 (95% CI 45.0–47.5) for the group treatment, resulting in a significant difference of 2.55 points (95% CI 1.61–3.49; *p* < 0.001). At 4-month follow-up, the two groups did not differ in their mean PCL-5 scores [difference 0.37 (95% CI −0.86 to 1.60); *p* = 0.551, Table 2]. Both groups showed a more than 10-point reduction in the mean PCL-5 score from admission to discharge and a more than 8-point reduction from admission to 4-month follow-up (Table 2). The results are visualized in Figure 2.

**Table 2.** Group differences in PTSD treatment outcomes^a^^,^^b^

	PCL-5 Score mean (95% CI)			Within-group PCL-5 score difference (95% CI) from admission	
Treatment	Admission	Discharge	Follow-up	Discharge	Follow-up
Group	60.0 (58.8–61.2)	46.2 (45.0–47.5)	51.5 (50.1–52.8)	−13.76 (−14.41 to −13.11)	−8.56 (−9.46 to −7.67)
Individual	61.2 (60.1–62.4)	43.7 (42.5–44.9)	51.1 (49.8–52.4)	−17.53 (−18.10 to −16.96)	−10.15 (−10.92 to −9.39)
Between group PCL-5 Score difference	−1.22 (−2.08 to −0.36)^c^	2.55 (1.61–3.49)	0.37 (−0.86 to 1.60)^d^		

Abbreviations: PCL-5, Posttraumatic Stress Disorder Checklist for DSM-5; PE, Prolonged exposure.

a The presented data is from mixed-model analyses.

b All changes not specified otherwise were statistically significant (*p* < 0.001).

c *P* = 0.006.

d *P* = 0.551.

### Secondary outcome

Mean satisfaction with progress (range 0–4 points) did not differ between the two groups at admission [difference, 0.04 (95% CI −0.02 to 0.10); *p* = 0.171]. At discharge, veterans in the individual treatment group were more satisfied with their progress [difference, 0.16 (95% CI 0.09–0.23); *p* = 0.001]. A similar result, but of smaller magnitude was found at 4-months follow-up [difference, 0.10 (95% CI 0.01–0.19); *p* = 0.029]. There was no difference regarding satisfaction with the provided care [range 0–4 points, difference, −0.05 (95% CI −0.17 to 0.07); *p* = 0.342).

### Sensitivity analyses

The results of the two sensitivity analyses are shown in the supplement (online Supplementary Tables S1–S4). The comparative effectiveness of group *v.* individual treatment was in line with the results from the main analysis in all three sensitivity analyses. There were few differences in baseline characteristics between participants that had missing data at the 4-month follow-up compared to those who did not. Importantly, there was no difference regarding the amount of participants with missing data between the group CPT and individual CPT/PE groups (online Supplementary Table S5).

## Discussion

Reduction of PTSD severity at the end of treatment was slightly larger for individual CPT or PE compared to group CPT but did not differ at follow-up. The between-group difference at the end of treatment was 2.55 points on the PCL-5, in favor of the individually delivered treatment. However, the point estimate of this difference, as well as the lower bound of the 95% confidence interval (95% CI 1.61–3.49), was smaller than the minimal clinically important difference of 7.9 points on the PCL-5 (Stefanovics, Rosenheck, Jones, Huang, & Krystal, 2018), implying similar effectiveness for both treatments. Clinically meaningful reductions in PTSD severity from beginning to the end of treatment were reached in both treatment groups. However, PTSD symptoms increased from discharge to 4-month follow-up in both groups. Differences in satisfaction with treatment progress between the two treatment groups mostly matched differences in PTSD symptom severity with the individual treatment group reporting significantly higher treatment satisfaction at discharge and at 4-month follow-up. However, the difference at end of treatment [range of satisfaction 0–4; difference, 0.16 (95% CI 0.09–0.23)], as well as at 4-month follow-up [range of satisfaction 0–4; difference, 0.10 (95% CI 0.01–0.19)], was of very small magnitude. Both groups were similarly satisfied with the provided care at follow-up.

The finding that there was only a small difference in average PCL-5 scores between the two groups at the end of treatment and no difference at 4-month follow-up is surprising in light of previous findings. There are several possible reasons why differences in treatment effectiveness of group CPT and individual CPT or PE are less pronounced than differences in treatment efficacy, which is greater for the latter (Schwartze, Barkowski, Strauss, Knaevelsrud, & Rosendahl, 2019). First, although participants in a randomized controlled trial agree to be randomly assigned to treatment, they usually still have personal preferences regarding the treatment modalities. Thus, random assignment to a less preferred treatment can reduce treatment engagement and lead to worse outcomes (Macias et al., 2009; Marcus, Stuart, Wang, Shadish, & Steiner, 2012). Second, some traumatic experiences are attached to stigma (e.g. sexual trauma) and may therefore be more difficult to address in front of a group than in an individual treatment setting. Although access to individual and/or group treatment in the current study was subject to limitations at the different treatment sites, personal preferences of patients were considered whenever possible (Karlin et al., 2010). Hence, the higher rate of participants reporting military sexual trauma in the individual treatment group might be indicative of such personal preferences and treatment matching. Third, the strongest evidence for head-to-head efficacy of group and individual CPT was established in active-duty military personnel treated in an outpatient setting (Resick et al., 2017). An outpatient population and setting differ from the residential setting of the present study meaning that participants lived and regularly interacted with each other on the perimeter of the treatment site.

The size of the treatment effects from admission to discharge for individually delivered trauma-focused treatments was comparable to the effects found in randomized controlled trials (Foa et al., 2018; Mavranezouli et al., 2020; Resick et al., 2017). Notably, the real-world effectiveness of treatments is usually lower than the efficacy measured in clinical trials because randomized clinical trials are conducted in carefully controlled conditions and have restrictive inclusion criteria. Our findings underscore that trauma-focused treatment is not only efficacious in clinical trials but also similarly effective in clinical practice (Eftekhari et al., 2013; Maguen et al., 2021). Although the PCL-5 scores for both treatment groups decreased more than the minimal clinically important difference from admission to 4-month follow-up, the overall PCL-5 scores at follow-up were still around 50 points and therefore clearly above the threshold suggestive of probable PTSD [PCL-5 scores above 31–33 points; (Bovin et al., 2016). Hence, further enhancing the longer-term effectiveness of trauma-focused treatments should be an ongoing priority for the field (Weber et al., 2021).

Due to our findings demonstrating only a small difference in effectiveness between group and individual trauma-focused treatment at discharge and no difference at four-month follow-up, additional research is needed to determine the role of group CPT in the VA's treatment program for veterans with PTSD. Although trauma-focused group treatments have outperformed waitlists (Schwartze et al., 2019; Sloan et al., 2013), superiority over non-trauma-focused treatment could not be established in these studies. Moreover, individual trauma-focused treatment did not only outperform non-trauma-focused treatment (Asmundson et al., 2019) but was also superior to group-treatment in a head-to-head study (Resick et al., 2017). As outlined above, disregarding individual preference and factors that might hinder treatment in a group setting (e.g. the nature of the trauma) will likely limit effectiveness of group treatments. Thus, future randomized trials incorporating treatment preferences into their design are needed to determine whether group CPT might be a cost-effective alternative to individual trauma-focused treatment for some veterans and could help to increase the currently limited access to such specialized treatments.

This study's findings are subject to several limitations. First, the retrospective cohort design limits the internal validity because results might be confounded by factors not included in this study. For example, due to the current data collection procedures, drop-out rates could not be determined and the previously higher documented attrition rates in trauma-focused group treatments compared to individually delivered treatments (Schwartze et al., 2019) could have affected our results. Second, we assumed missing data were missing at random (i.e. missingness only depends on observed data and not on unobserved data). However, there is no method to verify that this assumption holds based on the observed data. We had slightly higher rates of missing data in the individual therapy participants than in group therapy participants at discharge (29% *v.* 25% at discharge) but comparable rates at follow-up (65% *v.* 66% at follow-up). Third, results at follow-up should be interpreted with caution given the large proportion of missing data at follow-up. The results at follow-up may be biased if the missingness is not at random (for example, if subjects with higher PTSD severity are more or less likely to provide a follow-up observation). Fourth, we did not have access to information about whether patients received psychopharmacological intervention during or after residential treatment, nor about mental health treatment engagement between discharge and follow up. The resulting residual confounding complicates the interpretation of our results and limits their generalizability. Fifth, veteran's with a high level of imminent risk of harm to self or others were not eligible for the RRTP and thus, our results may not be generalizable to Veterans with PTSD who also present with the most severe levels of suicidality. Sixth, data on safety outcomes were not collected and thus we were unable to determine the comparative effectiveness of the treatment groups regarding adverse outcomes. Seventh, the data used was not primarily collected for the purpose of this study and is therefore subject to various biases, for example treatment fidelity was not assessed objectively but was self-reported by the treating clinicians which likely introduced a reporting bias. Some of these limitations could be addressed in a patient preference clinical trial, in which participants are allowed choose their preferred modality of treatment. Future research should also aim to identify whether one of the two treatment modalities clearly outperforms the other among certain subgroups of veterans. Furthermore, the moderating effect of PTSD severity at baselines on treatment outcomes should be the subject of future research. Finally, the high external validity of our findings for the VA PTSD residential treatment setting due to the completeness of the cohort is a strength of our study. On the other hand, the residential setting might limit the generalizability of our findings to in- or outpatient settings and to non-veteran populations.

Overall, at the end of residential treatment, veterans who received individual CPT or PE reported a greater reduction of PTSD severity of 2.55 points on the PCL (95% CI 1.61–3.49) compared to veterans treated with group CPT. No difference in effectiveness between the two treatment modalities was found at 4-month post-discharge follow-up. This partly contrasts previous evidence demonstrating limited efficacy of group CPT in randomized controlled trials. In light of the present findings, future treatment effectiveness studies are needed to determine under which circumstances group CPT could be considered as a first-line and cost-effective treatment modality for veterans with PTSD.

## Supplementary material

For supplementary material accompanying this paper visit https://doi.org/10.1017/S0033291722001441. click here to view supplementary material

## Data sharing statement

Data are subject to legal restrictions on confidential data of the United States Department of Veterans Affairs (VA) and cannot be shared by the authors. Free access to the data can be requested from the VA, however, access might be denied. More information can be found online (https://www.virec.research.va.gov) or by contacting Dr Harpaz-Rotem or the VA research office at the West Haven VA Medical Center.
