# Supplementary material for: Comparative effectiveness of group v. individual trauma-focused treatment for posttraumatic stress disorder in veterans
Source: Psychol Med. 2022 Aug 12;53(10):4561–8. doi: 10.1017/S0033291722001441 (PMC10388318; doi:10.1017/S0033291722001441)
Supplement: Supplementary file 1 [file S0033291722001441sup001.docx]

**Comparative Effectiveness of Group vs Individual Trauma-Focused Treatment for Posttraumatic Stress Disorder in Veterans**

**SUPPLEMENT**

**Figure S1. Distribution of raw PCL-5 scores**

Legend: The PTSD Checklist for DSM-5 (PCL-5) score assesses the severity of PTSD (range, 0-80, higher scores indicate higher symptom burden). The individual participants PCL-5 score is shown as individual dots. The Boxplot indicate the 25-75 quartiles of the data and the thick line the median of the corresponding sample.

**Sensitivity Analysis I: Adjusted for baseline PCL-5 score**

For this sensitivity analysis, we added the PCL-5 score at admission as a covariate to the mixed model. The dependent variable was the PCL-5 scores at discharge and follow-up. Otherwise, this sensitivity analysis followed the exact same procedure as the main analysis. The results of this first sensitivity analysis are presented in Table S1 and S2.

|  | **PCL-5 Score Mean (95% CI)** | | | |  |  |
| --- | --- | --- | --- | --- | --- | --- |
| **Timepoint** | **No.** | **Group CPT** | **No.** | **Individual CPT/PE** | **Difference (95% CI)** | ***P* Value** |
| Admission | 2736 | 59.8 | 3737 | 59.8 |  |  |
| Discharge | 2135 | 44.4 (42.7-46.1) | 2754 | 42.1 (40.5-43.7) | 2.36 (1.21-3.51) | .001 |
| Follow-up | 966 | 49.6 (47.8-51.5) | 1342 | 49.4 (47.7-51.1) | 0.27 (-1.19-1.73) | .718 |

**Table S1. Between Group Differences PTSD Treatment Outcomes^a^**

Abbreviations: CPT, Cognitive Processing Therapy; PCL-5, Posttraumatic Stress Disorder Checklist for DSM-5; PE, Prolonged exposure.

^a^The presented data is from mixed-model analyses.

**Sensitivity Analysis II: CPT and group CPT only**

For this sensitivity analysis, we excluded participants that received prolonged exposure. Otherwise, this sensitivity analysis followed the exact same procedure as the main analysis. The characteristics of this new sample is outlined in Table S2. The results of this second sensitivity analysis are presented in Table S3 and S4.

**Table S2. Demographics of Participants by Treatment Condition**

|  | **Treatment Condition, No (**%**) of Patients^a^** | | |  |
| --- | --- | --- | --- | --- |
| **Variable** | **Overall**  **(N = 5509)** | **Group CPT**  **(n = 2847)** | **Individual CPT (n = 2662)** | ***P* Value** |
| Age, median (IQR), y | 44 (35, 55) | 42 (34, 54) | 45 (35, 55) | <.001 |
| Female gender | 696 (13) | 206 (7.2) | 490 (18) | <.001 |
| Race |  |  |  | <.001 |
| American Indian/Alaskan | 245 (4.4) | 134 (4.7) | 111 (4.2) |  |
| Asian | 67 (1.2) | 34 (1.2) | 33 (1.2) |  |
| Black | 1414 (26) | 689 (24) | 725 (27) |  |
| Other | 155 (2.8) | 71 (2.5) | 84 (3.2) |  |
| Pacific Islander | 76 (1.4) | 60 (2.1) | 16 (0.6) |  |
| White | 3552 (64) | 1859 (65) | 1693 (64) |  |
| Ethnicity |  |  |  | <.001 |
| Hispanic | 502 (9.1) | 321 (11) | 181 (6.8) |  |
| Non-Hispanic | 5007 (91) | 2526 (89) | 2481 (93) |  |
| Education, median (IQR), y | 13 (12, 15) | 13 (12, 14) | 13 (12, 15) | .063 |
| Baseline symptom severity, median (IQR) |  |  |  |  |
| GAD-7^b^ | 16.0 (12.0, 19.0) | 16.0 (12.0, 19.0) | 16.0 (12.0, 19.0) | .88 |
| PHQ-9^c^ | 18.0 (14.0, 22.0) | 18.0 (14.0, 22.0) | 18.0 (14.0, 22.0) | .68 |
| Combat trauma | 3995 (73) | 2262 (79) | 1733 (65) | <.001 |
| Additional trauma^d^ |  |  |  |  |
| Military Sexual Trauma | 1364 (25) | 467 (16) | 897 (34) | <.001 |
| Non-Military Sexual Trauma | 1077 (20) | 427 (15) | 650 (24) | <.001 |
| Vehicle accident | 2740 (50) | 1499 (53) | 1241 (47) | <.001 |
| Other accident | 1365 (25) | 775 (27) | 590 (22) | <.001 |
| Victim of violence | 1946 (35) | 962 (34) | 984 (37) | .014 |
| Natural disaster | 916 (17) | 479 (17) | 437 (16) | .68 |
| Other traumatic incident | 2896 (53) | 1533 (54) | 1363 (51) | .050 |
| None | 182 (3.3) | 107 (3.8) | 75 (2.8) | .051 |
| Therapy |  |  |  |  |
| CPT Individual | 2662 (48) | 0 (0) | 2662 (100) |  |
| CPT Group | 2847 (52) | 2847 (100) | 0 (0) |  |
| Program completion | 5258 (95) | 2729 (96) | 2529 (95) | .13 |
| Length of Stay, median (IQR), d | 50 (46, 59) | 49 (46, 57) | 51 (46, 60) | .34 |
| SUD Services | 3690 (67) | 1986 (70) | 1704 (64) | <.001 |

Abbreviations: CPT, Cognitive processing therapy; GAD-7, Generalized Anxiety Disorder - 7 item; PCL-5, Posttraumatic Stress Disorder Checklist for DSM-5; PHQ-9, Patient Health Questionnaire - 9 item; SUD, Substance abuse disorder.

^a^Percentages have been rounded and may not total 100.

^b^Scores range from 0 to 21, with higher scores indicating worse symptoms. There were missing data resulting in the following sample sizes for this item: Overall, n = 3841; Group CPT, n = 1907; Individual CPT n = 1934.

^c^Scores range from 0 to 27, with higher scores indicating worse symptoms. There were missing data resulting in the following sample sizes for this item: Overall, n = 3843; Group CPT, n = 1905; Individual CPT n = 1938.

^d^Multiple answers could be given.

|  | **PCL-5 Score Mean (95% CI)** | | | |  |  |
| --- | --- | --- | --- | --- | --- | --- |
| **Timepoint** | **No.** | **Group CPT** | **No.** | **Individual CPT** | **Difference (95% CI)** | ***P* Value** |
| Admission | 2736 | 60.0 (58.7-61.3) | 2563 | 61.5 (60.2-62.8) | -1.44 (-2.40, -0.48) | .004 |
| Discharge | 2135 | 46.3 (45.0-47.6) | 1985 | 44.1 (42.8-45.4) | 2.19 (1.15, 3.23) | .001 |
| Follow-up | 966 | 51.5 (50.0-53.0) | 929 | 51.2 (49.8-52.7) | 0.28 (-1.07, 1.63) | .685 |

**Table S3. Between Group Differences PTSD Treatment Outcomes^a^**

Abbreviations: CPT, Cognitive processing therapy; PCL-5, Posttraumatic Stress Disorder Checklist for DSM-5.

^a^The presented data is from mixed-model analyses.

**Table S4. Within Group Differences PTSD Treatment Outcomes^a,b^**

|  | **PCL-5 Score Mean (95% CI)** | | | **PCL-5 Score Difference (95% CI) from Admission** | |
| --- | --- | --- | --- | --- | --- |
| **Treatment** | **Admission** | **Discharge** | **Follow-Up** | **Discharge** | **Follow-Up** |
| Group | 60.0 (58.7-61.3) | 46.3 (45.0-47.6) | 51.5 (50.0-53.0) | -13.75 (-14.40, -13.10) | -8.55 (-9.45, -7.65) |
| Individual | 61.5 (60.2-62.8) | 44.1 (42.8-45.4) | 51.2 (49.8-52.7) | -17.38 (-18.05, -16.71) | -10.27 (-11.19, -9.35) |

Abbreviations: CPT, Cognitive processing therapy; PCL-5, Posttraumatic Stress Disorder Checklist for DSM-5.

^a^The presented data is from mixed-model analyses.

^b^All changes were statistically significant (*P* < .001)

**Table S5. Characteristics of Participants Lost to Follow-Up**

|  | **Lost to Follow-Up, No (**%**) of Patients^a^** | | |  |
| --- | --- | --- | --- | --- |
| **Variable** | **Overall**  **(N = 6735)** | **No**  **(n = 2308)** | **Yes (n = 4427)** | ***P* Value** |
| Age, median (IQR), y | 44 (35, 55) | 47 (37, 57) | 41 (34, 53) | <.001 |
| Women (gender) | 859 (13) | 312 (14) | 547 (12) | .38 |
| Race |  |  |  | .011 |
| American Indian/Alaskan | 303 (4.5) | 94 (4.1) | 209 (4.7) |  |
| Asian | 77 (1.1) | 23 (1.0) | 54 (1.2) |  |
| Black | 1,730 (26) | 639 (28) | 1091 (25) |  |
| Other | 197 (2.9) | 70 (3.0) | 127 (2.9) |  |
| Pacific Islander | 82 (1.2) | 17 (0.7) | 65 (1.5) |  |
| White | 4346 (65) | 1465 (63) | 2881 (65) |  |
| Ethnicity |  |  |  | .94 |
| Hispanic | 592 (8.8) | 202 (8.8) | 390 (8.8) |  |
| Non-Hispanic | 6143 (91) | 2106 (91) | 4037 (91) |  |
| Education, median (IQR), y | 13 (12, 14) | 13 (12, 15) | 13 (12, 14) | .51 |
| Baseline symptom severity, median (IQR) |  |  |  |  |
| PCL-5^b^ | 61 (53, 68) | 61 (52, 68) | 61 (53, 68) | .019 |
| GAD-7^c^ | 16.0 (12.0, 19.0) | 16.0 (12.0, 19.0) | 16.0 (13.0, 19.0) | .052 |
| PHQ-9^d^ | 18.0 (14.0, 22.0) | 18.0 (14.0, 22.0) | 18.0 (14.0, 22.0) | .17 |
| Combat trauma | 4892 (73) | 1609 (70) | 3283 (74) | <.001 |
| Additional trauma^e^ |  |  |  |  |
| Military Sexual Trauma | 1680 (25) | 621 (27) | 1059 (24) | .007 |
| Non-Military Sexual Trauma | 1286 (19) | 434 (19) | 852 (19) | .66 |
| Vehicle accident | 3320 (49) | 1124 (49) | 2196 (50) | .48 |
| Other accident | 1659 (25) | 542 (23) | 1117 (25) | .11 |
| Victim of violence | 2319 (34) | 761 (33) | 1558 (35) | .069 |
| Natural disaster | 1089 (16) | 386 (17) | 703 (16) | .37 |
| Other traumatic incident | 3537 (53) | 1200 (52) | 2337 (53) | .53 |
| None | 229 (3.4) | 76 (3.3) | 153 (3.5) | .73 |
| Therapy |  |  |  | .62 |
| Group | 2847 (42) | 966 (42) | 1881 (42) |  |
| Individual | 3888 (58) | 1342 (58) | 2546 (58) |  |
| Program completion | 6330 (95) | 2182 (96) | 4148 (95) | .23 |
| Length of Stay, median (IQR), d | 51 (46, 60) | 51 (46, 58) | 51 (46, 62) | <.001 |
| SUD Services | 4486 (69) | 1451 (64) | 3035 (70) | <.001 |

Abbreviations: CPT, Cognitive processing therapy; GAD-7, Generalized Anxiety Disorder - 7 item; PCL-5, Posttraumatic Stress Disorder Checklist for DSM-5; PHQ-9, Patient Health Questionnaire - 9 item; SUD, Substance abuse disorder.

^a^Percentages have been rounded and may not total 100.

^b^Scores range from 0 to 80, with higher scores indicating worse symptoms. There were missing data resulting in the following sample sizes for this item: Overall, n = 6473; Not lost, n = 2197; Lost n = 4276.

^c^Scores range from 0 to 21, with higher scores indicating worse symptoms. There were missing data resulting in the following sample sizes for this item: Overall, n = 4684; Not lost, n = 1564; Lost n = 3120.

^d^Scores range from 0 to 27, with higher scores indicating worse symptoms. There were missing data resulting in the following sample sizes for this item: Overall, n = 4678; Not lost, n = 1553; Lost n = 3125.

^e^Multiple answers could be given.
